# Gegen Qinlian Decoction combined with Metformin for the treatment of patients with Type-2 Diabetes Mellitus: A retrospective observational study

**DOI:** 10.12669/pjms.39.4.7776

**Published:** 2023

**Authors:** Yingqi Chen, Xiazhi Zhou, Zixiang Jiang, Yinglian Liu

**Affiliations:** 1 Yingqi Chen Department of Chinese Medicine Prescription, Hainan Medical University, Haikou 571199, Hainan Province, P.R. China; 2 Xiazhi Zhou Department of Gynecology of Traditional Chinese Medicine, Hainan Medical University, Haikou 571199, Hainan Province, P.R. China; 3 Zixiang Jiang School of Traditional Chinese Medicine. Hainan Medical University, Haikou 571199, Hainan Province, P.R. China; 4 Yinglian Liu Department of Gynecology of Traditional Chinese Medicine, Hainan Medical University, Haikou 571199, Hainan Province, P.R. China

**Keywords:** Gegen Qinlian Decoction, GQD, Metformin, Type-2 diabetes mellitus, T2DM, Traditional Chinese Medicine

## Abstract

**Objectives:**

To investigate Gegen Qinlian Decoction (GQD) combined with metformin for treatment of patients with Type-2 Diabetes Mellitus (T2DM).

**Methods:**

This retrospective observational study reviewed the clinical data of 89 patients diagnosed with T2DM in the Department of Acupuncture and Massage, Hainan Medical University from January 2021 to June 2022. Patients were non-randomized and divided into two groups based on the treatment received: observation group (n=41, GQD combined with metformin); control group (n=48, metformin only). Fasting blood glucose levels (FBG), traditional Chinese medicine (TCM) syndrome scores, clinical effect, blood glucose time in range and adverse reactions were compared between the two groups.

**Results:**

There were no statistically significant differences in age, gender, BMI and duration of T2DM between the two groups (*P*>0.05). The FBG, 2h glucose, HbA1c levels and TCM syndrome scores of the two groups were significantly lower post-treatment (*P*<0.001) with a greater decrease in the observation group (*P*<0.001). The observation group was more clinically efficacious than the control group post-treatment (92.68% *vs*. 77.08%; *P*<0.05). Blood glucose time in range and the incidence of adverse reactions were lower in the observation group than the control group (*P*<0.001 and *P*<0.05).

**Conclusions:**

GQD combined with metformin can significantly reduce FBG, 2h glucose and HbA1c levels, and improve TCM syndrome, with good clinical efficacy, shorter blood glucose time in range and less adverse reactions.

## INTRODUCTION

Diabetes mellitus (DM) is the ninth leading cause of death worldwide and more than one million deaths are attributed to DM each year.^1^prevalence, and burden of suffering of diabetes mellitus based on epidemiological data from the Global Burden of Disease (GBD Type-2 diabetes mellitus (T2DM) accounts for 90% of all DM cases.^2^ It is estimated that the global cost of diabetes is set to nearly double to $2.5 trillion by 2030.^3^the future economic consequences of the disease remain opaque. We forecast the full global costs of diabetes in adults through the year 2030 and predict the economic consequences of diabetes if global targets under the Sustainable Development Goals (SDG Therefore, T2DM is a serious public health problem that not only has a huge impact on individual health, but also imposes a considerable economic burden on individuals and society. Traditional Chinese medicine (TCM) believes that diabetes is mainly characterized by “spleen rash” symptoms such as obesity, sweet and greasy mouth, abdominal distension, sleepiness or diarrhea in the early stages, and the core pathogenesis is “phlegm dampness, dryness and heat”, so it is called damp-heat T2DM in TCM.^4^,^5^ Gegen Qinlian Decoction (GQD), a classic TCM formula, is widely used in the treatment of T2DM in China.^6^ Studies have found that GQD can greatly reduce fasting blood glucose (FBG), glycated hemoglobin A1c (HbA1c), glycosylated serum protein and fasting insulin in diabetic rats.^7^,^8^

The main active ingredients of GQD, including puerarin, baicalin, berberine and glycyrrhizin, have been shown to have hypoglycemic effects.^9^,^10^ Metformin has been recommended as the first-line glucose-lowering treatment for T2DM and is effective both as monotherapy and in combination with other hypoglycemic medications.^11^ There are a number of studies on the role of metformin combined with other antihyperglycemic agents on T2DM, but there is limited research on the combination of metformin and GQD.^12^,^13^ This study aimed to investigate the clinical effect of GQD combined with metformin for treatment of patients with T2DM to provide evidence for clinical treatment.

## METHODS

In this retrospective observational study, the clinical data of 89 patients diagnosed with T2DM in the Department of Acupuncture and Massage, Hainan Medical University from January 2021 to June 2022 were reviewed. Patients were nonrandomized and divided into two groups based on the treatment received. Patients who received metformin only were assigned to the control group (n=48), and patients who received GQD combined with metformin were assigned to the observation group (n=41)

### Ethical Approval:

The study was approved by the ethics committee of Hainan Medical College (No. HYLL-2022-134, Date: 2022-03-24), and written informed consent was obtained from all patients.

### Inclusion Criteria:

- Patients between the ages of 18 and 70 years.
- Diagnosed with T2DM: FPG ≥7.0 mmol/L or 2h plasma glucose (2h PG) during oral glucose tolerance test (OGTT) ≥11.1 mmol/L.^14^
- Identified with damp-heat type T2DM.^4^,^5^
- With complete clinical data.

### Exclusion Criteria:

- Patients with Type-1 diabetes mellitus.
- With severe mental disorders.
- With severe liver and kidney dysfunction.
- With contraindications to metformin.
- Complicated with malignant tumor.
- Were pregnant and lactating women.

The study followed the Strengthening the Reporting of Observational Studies in Epidemiology (STROBE) reporting guideline (https://www.strobe-statement.org/).^15^

### Treatment Methods:

### Control group:

Patients were treated with metformin (Beijing Jingfeng Pharmaceutical Group Co., Ltd.; approval no.: H11021518; specification: 0.25g/piece) twice a day at a dose of 500mg. The dose was adjusted according to the blood glucose monitoring of the patients and controlled within 2000 mg/d.^16^ The treatment lasted two months with daily dietary guidance and lifestyle intervention.

### Observation group:

Patients were treated with GQD combined with metformin. Metformin treatment was the same as the control group. The formula of GQD was Pueraria lobata (Willd.) Ohwi (ge gen) 15g, Scutellaria baicalensis Georgi (huang qin) 9g, Coptidis chinensis Franch. (huang lian) 9g and Glycyrrhiza uralensis Fisch. (gan cao) 6g.^17^ If the patient had weakness of limbs and abnormal sweating, calcined keel and floating wheat were added. If the patient had dry stool, dry mouth, and bad breath, heterophylla, Radix Ophiopogon japonicus, and Schisandra were added. If the patient was physically weak, tired, or sleepless at night, Suanzaoren and polygala were added.^14^ The decoction was decocted with water for 200mL in total/bag by the TCM pharmacy department of our hospital. The patients were treated with the decoction twice a day, once in the morning and once in the evening, 200mL each time, continuously for two months with daily dietary guidance and lifestyle intervention.

### Observational Indicators:

Blood glucose levels: FPG, 2-h PG and HbA1c of all patients pre- and post-treatment were collected.

### Traditional Chinese medicine (TCM) syndrome scores:

A TCM syndrome scale was adopted to evaluate the TCM syndrome scores of the patients pre- and post-treatment by physicians. The main symptoms assessed included distension and fullness in the abdomen, thirst and lack of drinking, lack of appetite, and head and body fatigue. Scores were 0, 2, 4, and 6 points, and the higher the score, the more serious the syndrome. Secondary symptoms included limb fatigue, heart and chest congestion, red and yellowish urine, and uncomfortable bowel movements and were scored as 0, 1, 2, and 3 points, with higher scores being more serious.^5^ Total TCM syndrome score = main symptom score + secondary symptom score.

### Clinical effect assessment is described below.^18^

1. ***Markedly effective:*** Disappearance of clinical symptoms post-treatment, FPG <7mmol/L, and two hours PG <8.3mmol/L.
2. ***Effective:*** Significant improvement in clinical symptoms post-treatment, FPG 7-9mmol/L, and two hours PG 8.3-10.5mmol/L.
3. ***Ineffective:*** No improvement in clinical symptoms post-treatment, FPG >9mmol/L, and 2h PG >10.5mmol/L.

Total effective rate = (Number of markedly effective patients + Number of effective patients) / Total number of patients × 100%.

Blood glucose time in range and adverse reactions: Clinical data about blood glucose time in healthy range and adverse reactions post-treatment were collected. Adverse reactions included diarrhea, nausea and vomiting, and hypoglycemia.

### Statistical Analysis:

Data were analyzed using SPSS 22.0 (IBM, USA). Continuous, normally distributed variables were presented as mean ± SD, and Student’s t-test was used to compare the differences between the two groups. Continuous, not normally distributed variables were presented as median (interquartile range), and Mann-Whitney U-test was used to compare the differences between the two groups. Paired-sample t-test compared before and after treatment within a group. Categorical variables were presented as frequency and percentage (n, %), and Chi-square test was used to compare the differences between the two groups. The differences were considered statistically significant when *P*<0.05.

## RESULTS

The observation group consisted of 24 males and 17 females, aged 42-70 years, and the control group consisted of 26 males and 22 females, aged 43-67 years (*P*=0.831). There were no statistically significant differences in age (53.66±6.84 *vs*. 53.13±5.24), BMI (26.51 (IQR range: 25.25-27.23) *vs*. 26.58 (IQR range: 25.29-27.00) and duration of T2DM (5.00(IQR range: 4.00-6.00) *vs*. 5.00 (IQR range: 4.00-6.00)) between the observation or control groups (*P*>0.05).

Pre-treatment, there were no statistically significant differences in FBG, 2h PG and HbA1c levels between the two groups (*P*>0.05). Post-treatment, FBG, 2h PG and HbA1c levels of the two groups decreased significantly (*P*<0.001) with a greater decrease in the observation group (*P*<0.001; Table-I). Pre-treatment, there were no statistically significant differences in the TCM syndrome scores between groups (*P*>0.05). TCM syndrome scores were significantly lower post-treatment (*P*<0.001) with a greater decrease in the observation group (*P*<0.001; Table-II).

**Table-I:** Blood glucose levels pre- and post-treatment.

	FBG (mmol/L)		2h PG (mmol/L)		HbA1c (%)	

	Pre-treatment	Post-treatment	Pre-treatment	Post-treatment	Pre-treatment	Post-treatment
Observation group (n=41)	8.37±1.38	5.43±1.02*	10.24±0.90	7.79±0.84*	8.35±0.51	5.23±0.42*
Control group (n=48)	8.42±1.29	6.73±1.19*	10.16±0.86	8.57±0.81*	8.37±0.56	5.99±0.56*
*t*	-0.187	-5.483	0.389	-4.424	-0.191	-7.182
P	0.852	<0.001	0.698	<0.001	0.849	<0.001

* , P<0.001 when compared with the same group pre-treatment.

**Table-II:** TCM syndrome scores pre- and post-treatment [mean (IQR range)].

	Pre-treatment	Post-treatment
Observation group (n=41)	23.00(22.00-25.00)	13.00(11.50-15.00)*
Control group (n=48)	23.50(22.25-24.75)	16.00(15.00-17.75)*
*Z*	-0.664	-5.421
*P*	0.507	<0.001

*, P<0.001 when compared with the same group pre-treatment.

The clinical effective rate in the observation group (92.68%) was higher than in the control group (77.08%) post-treatment (*P*<0.05; Table-III). Blood glucose time in range and the incidence of adverse reactions were significantly lower in the observation group (*P*<0.001; Table-IV).

**Table-III:** The clinical effect between the two groups (n, %).

	Markedly effective	Effective	Ineffective	Total effective rate
Observation group (n=41)	24(58.54)	14(34.15)	3(7.31)	38(92.68)
Control group (n=48)	16(33.33)	21(43.75)	11(22.92)	37(77.08)
*χ^2^*				7.065
*P*				<0.05

**Table- IV:** Blood glucose time in range and adverse reactions post-treatment.

	Blood glucose time in range (day, mean ± SD)	Adverse reactions (frequency, %)			

		Hypoglycemia	Diarrhea	Nausea/vomiting	Adverse reaction rate
Observation group (n=41)	8.07±1.79	2(4.88)	1(2.44)	2(4.88)	5(9.80)
Control group (n=48)	14.79±2.37	7(14.58)	6(12.50)	6(12.50)	19(39.58)
*t*/*χ2*	-14.870				8.422
*P*	<0.001**				<0.05*

* , Chi-square test;

** , Student’s *t*-test.

## DISCUSSION

In this study, the combination of GQD and metformin showed a good efficacy in reducing blood glucose time in range and adverse reactions. Although metformin has been recommended as the first-line hypoglycemic agent for T2DM, studies have shown that combined therapy of metformin and other medications is more efficacious than metformin monotherapy.^14^,^19^ It was reported that long-term administration of metformin may lead to insulin resistance (IR), gastrointestinal side effects, lactic acidosis, and vitamin B12 deficiency.^20^ Thus there is a need for a safer and efficacious treatment for T2DM. In TCM, diabetes is a “Xiaoke disease (a disease characterized by frequent drinking and urination)”, which is considered to be caused by “Yin deficiency and dryness-heat”.^21^ TCM believes that the treatment of “Yin deficiency and dryness-heat” requires the circulation of Yang, the nourishment of Qi and Yin, and the elimination of blood stasis, reducing blood glucose levels and ing dampness and heat.^21^ In our study, GQD consisted of Pueraria lobata (Willd.) Ohwi (ge gen), Scutellaria baicalensis Georgi (huang qin), Coptidis chinensis Franch. (huang lian) and Glycyrrhiza uralensis Fisch. (gan cao), which can remove dampness and heat. Pueraria lobata (Willd.) Ohwi (ge gen) can clear away heat by Yangming meridian and produce fluid to moisten dryness.^22^ Scutellaria baicalensis Georgi (huang qin) and Coptidis chinensis Franch. (huang lian) can clear away excess heat from the stomach and lung, which could remove glucose from the blood.^23^

Studies have also reported the hypoglycemic effect of Pueraria lobata (Willd.) Ohwi (ge gen), Scutellaria baicalensis Georgi (huang qin) and Coptidis chinensis Franch. (huang lian).^20^ Ryuk et al reported that when combined with metformin, GQD had a synergistic effect on blood glucose control and could effectively improve diabetes-related clinical symptoms including sweating, thirst, fear of heat, and other related symptoms.^24^ Since GQD is a combination of several Chinese herbs, the effects of GQD are multi-factorial with complex mechanistic effects in T2DM, but Tu et al. found that the anti-diabetic/or antihyperglycemic effects of GQD worked by regulating adipocytic PPARα and PPARγ signaling systems to maintain glucose and lipid metabolisms.^23^ In this study, it was found that the combination of GQD and metformin can significantly reduce FBG, 2h PG, HbA1c levels and improve TCM syndrome, which supported the findings by Chen et al.^25^

It was also found that the combination of GQD and metformin can reduce blood glucose time in range and adverse reactions. Hypoglycemia, nausea, vomiting and diarrhea are the most common adverse reactions of metformin.^18^ The results of the study showed less adverse reactions in the observation group than the control group, suggesting GQD plus metformin has better safety. GQD is traditionally used for diarrhea and dysentery in TCM. In animal experiments, it was reported that GQD treats diarrhea by regulating gut microbiota and short-chain fatty acids.^20^

### Limitations of the study:

This was a retrospective study with a small sample size, possibly limiting the extrapolation of the results. The course of treatment was short and studies on long-term efficacy of GQD combined with metformin in patients with T2DM should be carried out in the future. Although TCM syndrome was evaluated by professionally trained doctors, there is still subjectivity, which may make the results biased.

## CONCLUSION

GQD combined with metformin can significantly reduce FBG, 2h PG and HbA1c levels, and improve TCM syndrome, with good clinical efficacy, shorter blood glucose time in range and less adverse reactions in patients with T2DM.

### Authors’ contributions:

**YC** and **XZ**: Conceived, designed the study, involved in the writing of the manuscript and are responsible for the integrity of the study.

**YC**, **XZ**, **ZJ**, and **YL:** Collected the data and performed the analysis.

All authors have read and approved the final manuscript.
